# Cervical spondylosis with propriospinal myoclonus and vocalization: a case report

**DOI:** 10.3389/fsurg.2026.1714513

**Published:** 2026-03-31

**Authors:** Jin-Hui Peng, Bo-Wei Liang, Xiang-Hong Zeng, Zong-Quan Huang, Shu-Heng Zhou, Yi-Sheng Wang, Chun-Feng Lai, Cheng-Zhen Pan

**Affiliations:** 1 Yulin Orthopedic Hospital of Integrated Traditional Chinese and Western Medicine, Yulin, Guangxi Zhuang Autonomous Region, China; 2 First People’s Hospital of Yulin City, Yulin, Guangxi Zhuang Autonomous Region, China

**Keywords:** case report, cervical decompression, cervical spondylosis, involuntary vocalization, propriospinal myoclonus

## Abstract

**Background:**

Cervical spondylosis typically presents with neck pain, radiculopathy, or myelopathy, but rarely with spinal myoclonus. Propriospinal myoclonus (PSM)—characterized by involuntary trunk and limb jerks—is an exceedingly rare manifestation of cervical spondylosis, with limited reports of associated vocalizations.

**Case presentation:**

A 55-year-old man presented with a 3-year history of neck pain, which progressed to dizziness, lower limb weakness, and intermittent propriospinal myoclonus manifesting as hand tremors and involuntary vocalizations. Cervical MRI and CT revealed C3/4–C5/6 disc protrusion, C5 instability, and spinal cord compression. Initial conservative treatments were ineffective. Endoscopic anterior cervical decompression and fusion (C3–C6) was performed, removing protruding discs and relieving spinal cord compression. Postoperatively, myoclonus and vocalizations resolved within 24 h, with improved lower limb strength and gait stability (mJOA: 12 → 18). Imaging confirmed restored cervical lordosis and implant stability. No recurrence was observed at 12-month follow-up.

**Conclusions:**

This rare case of cervical spondylosis presenting with PSM and vocalizations highlights the diagnostic challenge of distinguishing spinal myoclonus from epilepsy or psychogenic disorders. Endoscopic anterior cervical decompression effectively alleviated symptoms, suggesting a compressive etiology. Clinicians should consider spinal myoclonus in atypical presentations of cervical spondylosis, and early surgical intervention may be warranted in refractory cases. This report underscores the role of comprehensive imaging and multidisciplinary evaluation in managing complex spinal disorders.

## 1 Background

Cervical spondylosis is a degenerative condition affecting cervical intervertebral discs and adjacent structures, leading to nerve and vessel pathology and presenting with imaging-related symptoms (1). In China, its prevalence is approximately 13.76%, and globally, it is prevalent in adults over 50, representing a leading cause of disability (2). It is classified by affected tissues into cervical, radicular, myelopathic, vertebral artery, and sympathetic types. Typical symptoms include neck pain, limb numbness, unsteady gait, dizziness, and palpitations. Rarely, spinal cord compression triggers involuntary limb or trunk jerks, and even less commonly, abdominal myoclonus from cervical spondylosis causes involuntary vocalizations. In 1974, Hopkins described myoclonus from focal spinal compression as “spinal myoclonus (SM).” Cervical spondylosis manifesting as SM—particularly propriospinal myoclonus (PSM) with involuntary vocalizations—is extremely rare and often prone to misdiagnosis as epilepsy or psychogenic disorders due to associated psychiatric symptoms, with unclear treatment strategies (3). We report a case of cervical spondylosis with PSM successfully treated via minimally invasive anterior cervical endoscopic decompression and fusion. This case highlights the risks of misdiagnosis (e.g., psychogenic disorders), the compressive mechanisms underlying PSM, and the benefits of endoscopic techniques (e.g., minimal tissue damage, rapid recovery), offering insights into the management of complex spinal disorders.

## 2 Case presentation

On 14 November 2023, a 55-year-old man presented with a 3-year history of cervical pain, which had worsened over the past month and was accompanied by dizziness, lower limb weakness, and PSM. Three years earlier, he had developed intermittent cervical pain, aggravated by activity and relieved by rest, but unresponsive to multiple conservative treatments. One month before admission, his symptoms intensified, with dizziness, lower limb weakness, PSM-induced hand tremors, and involuntary vocalizations (approximately 2 episodes per day, lasting 5–15 min, worse before sleep). These episodes were potentially linked to abdominal myoclonus and occasional abdominal discomfort. His past medical history included treated secondary pulmonary tuberculosis and sleep disturbances. He had previously sought care at multiple institutions (e.g., First Naval Hospital of the Southern Theater Command) and was diagnosed with cervical spondylosis; however, treatments were unsuccessful. He denied having diabetes, hypertension, or a history of stroke. His initial Visual Analog Scale (VAS) score was approximately 6, indicating moderate pain.

**Figure 1:**
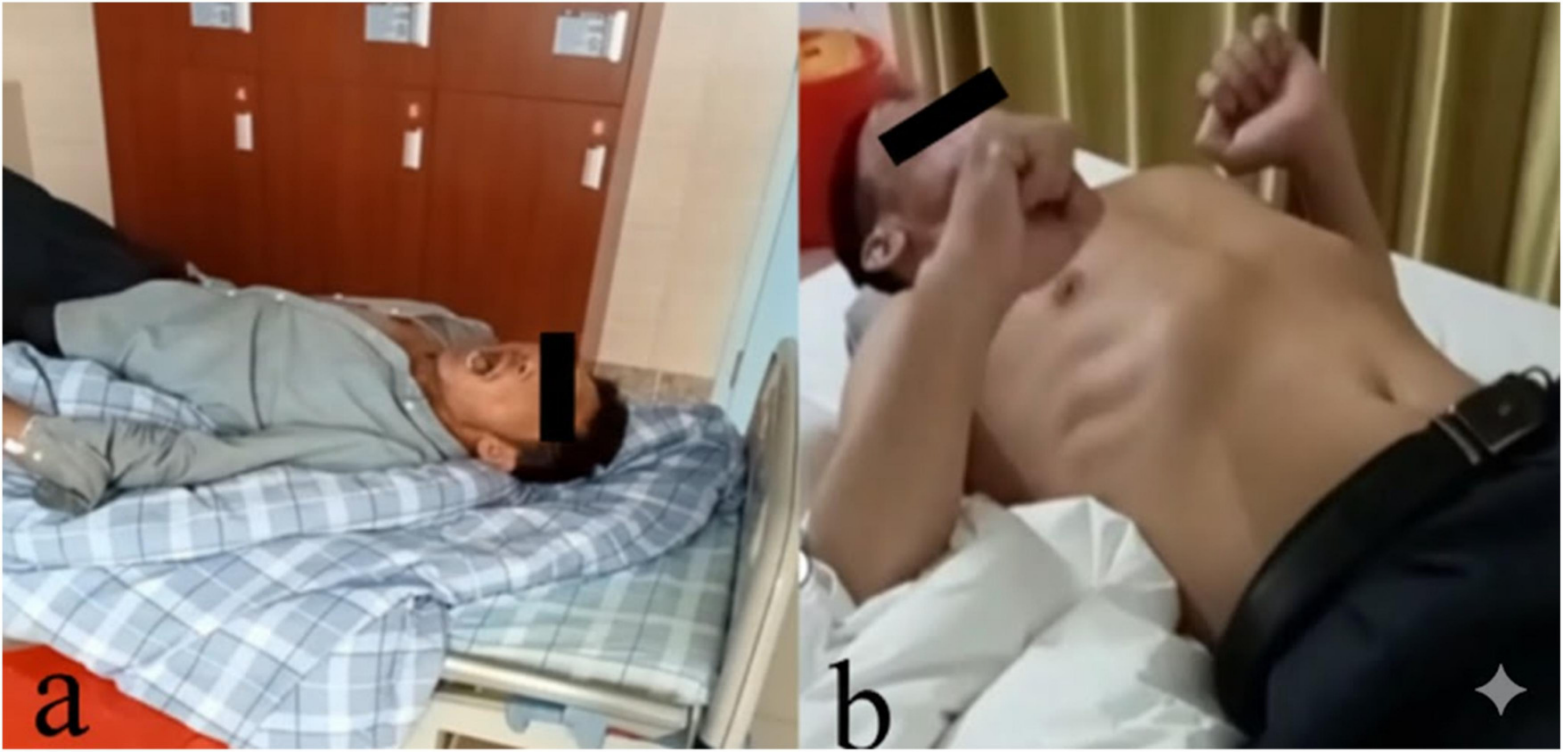
Physical examination. **(a)** The patient displays involuntary and unusual vocalizations, accompanied by a pained expression; and **(b)** the patient experiences occasional convulsions characterized by tightly clenched hands.

**Figure 2:**
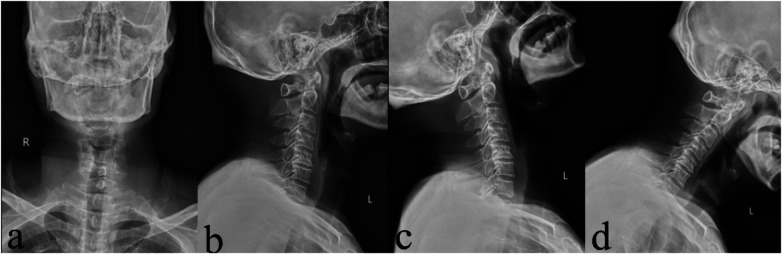
Preoperative cervical spine DR. **(a)** Anteroposterior view; **(b)** lateral view; **(c)** hyperextension view; and **(d)** hyperflexion view.

**Figure 3:**
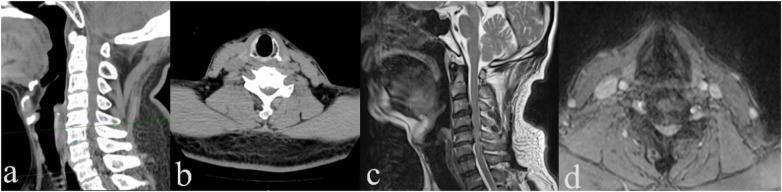
Preoperative cervical spine CT and MRI. **(a)** Cervical spine CT sagittal view; **(b)** cervical spine CT axial view at C5/6; **(c)** cervical spine MRI sagittal view; and **(d)** cervical spine MRI axial view at C5/6.

The physical examination revealed mild straightening of the cervical lordosis, slight neck tenderness, and normal range of motion. Muscle strength was grade 5 in major bilateral upper and lower limb groups, but increased upper limb spasticity was observed. Hyperreflexia was present in bilateral biceps, triceps, knees, and Achilles tendons, with positive patellar and ankle clonus and Hoffmann's sign. Oppenheim, Chaddock, Babinski, and Gordon signs were negative (Figures 1a,b). Laboratory tests revealed hepatitis B surface antibody 45.83 mIU/mL, core antibody 15.11 PEIU/mL, alanine aminotransferase 74 U/L, aspartate aminotransferase 53 U/L, and serum potassium 3.45 mmol/L, possibly related to prior pulmonary tuberculosis. Imaging revealed C5 vertebral instability, C3/4, C4/5, and C5/6 disc protrusion with spinal canal stenosis, thecal sac compression, and resulting obstructed cerebrospinal fluid flow, correlating with PSM symptoms (e.g., two episodes/day) (Figures 2,3). Cranial CT and MRI were normal.

**Figure 4:**
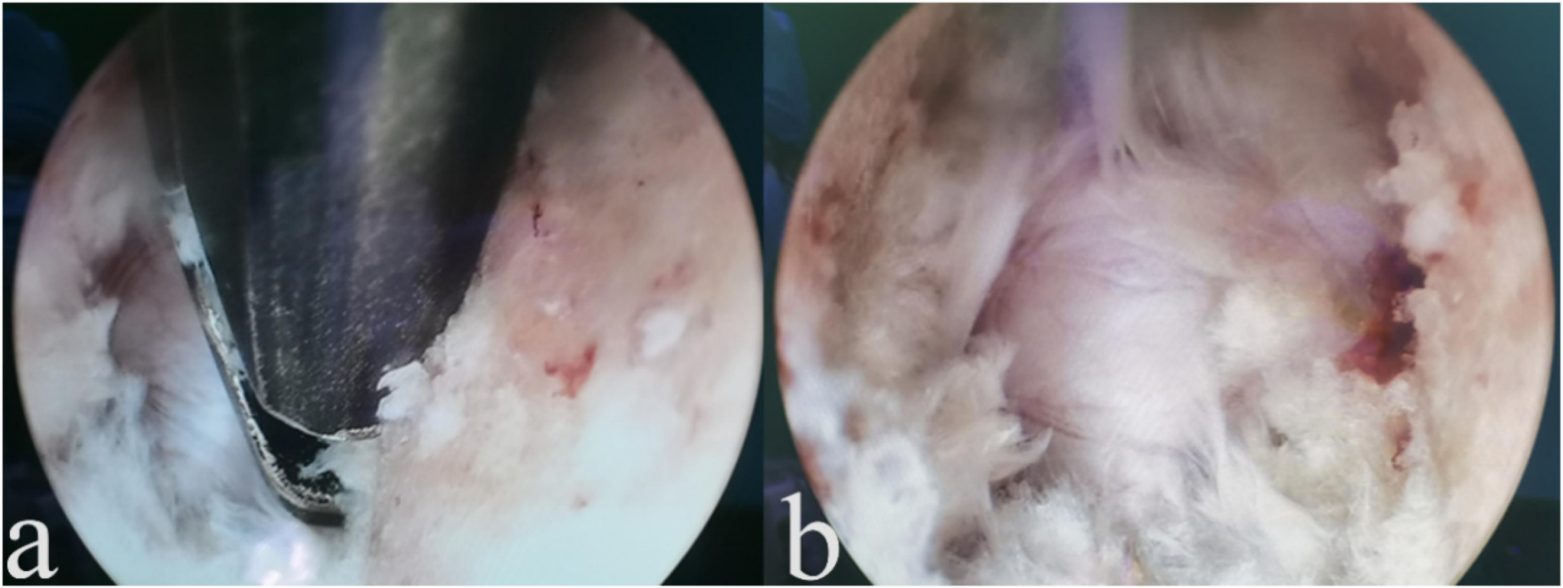
Intraoperative procedures. **(a)** Debridement of the posterior longitudinal ligament and hypertrophic tissue; and **(b)** exposure of the dural sac.

Following comprehensive evaluation, including both MRI and CT that demonstrated C3/4–C5/6 disc protrusion and thecal sac compression, we diagnosed the patient with cervical myelopathy accompanied by PSM. On 22 November 2023, an anterior endoscopic cervical discectomy and fusion (ACDF) was performed at C3–C6 under general anesthesia. The patient was positioned supine with elevated shoulders and a neutral neck. A 6-cm oblique incision was made along the medial border of the right sternocleidomastoid to expose the anterior cervical spine. Performing this multilevel (C3–C6) decompression via a minimally invasive approach presented significant technical challenges, particularly in maintaining optimal visualization across three motion segments through a limited surgical corridor. C-arm fluoroscopy localized the C3–C6 interspaces, and positioning pins were used to open the spaces. We excised anterior osteophytes and annulus, introduced the Joimax TESSYS endoscopic system (3.7-mm working channel) with saline irrigation, removed protruding discs, incised the posterior longitudinal ligament, and decompressed the spinal cord and nerve roots (4, 5). Zero-profile fusion cages were specifically selected for this multilevel reconstruction to minimize the risk of postoperative dysphagia, which is frequently associated with traditional plate-and-screw constructs in long-segment anterior surgeries (6, 7). C-arm confirmed optimal placement. Blood loss was approximately 50 mL, vital signs remained stable, and neurological function improved immediately postoperatively (Figure 4).

**Figure 5:**
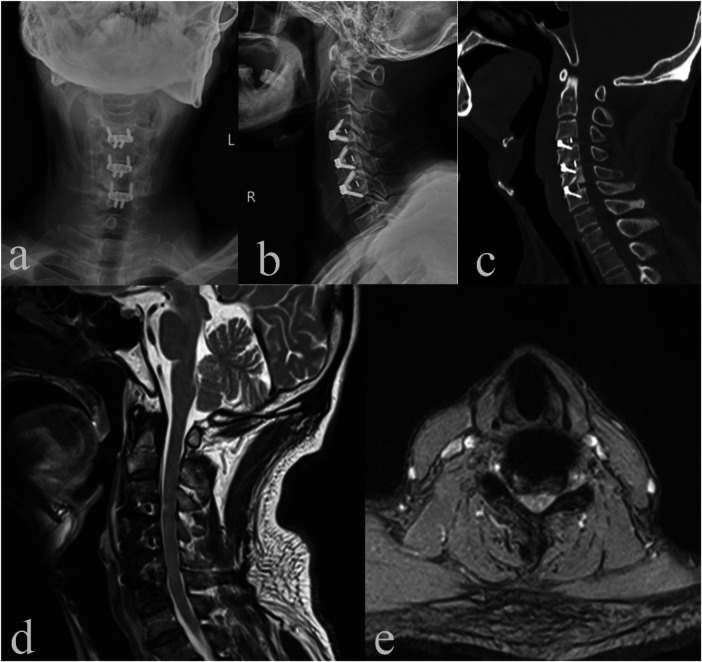
Postoperative cervical spine DR, CT, and MRI. **(a)** Cervical spine DR anteroposterior view; **(b)** cervical spine DR lateral view; **(c)** cervical spine CT sagittal view; **(d)** cervical spine MRI sagittal view; and **(e)** cervical spine MRI axial view at C5/6.

Postoperative imaging confirmed restored lordosis, stable implants, expanded canal, and relief of thecal sac compression (Figure 5), directly correlating with the resolution of both myoclonus and vocalizations. At discharge, both myoclonus and involuntary vocalizations had resolved, with notable recovery in lower limb strength and gait (VAS score decreased from 6 to 1), and both mJOA and Tinetti scores showed significant enhancement (Table 1). At the 12-month follow-up, gait had normalized, daily activities remained unimpaired, and no symptoms recurred. The patient resumed manual labor. Involuntary vocalizations were likely due to abdominal myoclonus.

**Table 1:** Pre- and postoperative recovery of clinical symptoms in patients.

Assessment index	Preoperative	1 Month postoperative	6 Months postoperative	18 Months postoperative
mJOA mJOA score (points)	12	16	18	18
Tinetti Tinetti score (points)	16	22	26	27
Myoclonic seizure frequency	2 times/day	times/day 0	0	0
Crying out episodes	2 times/day	times/day 0	0	0

## 3 Discussions

### 3.1 Clinical rarity and diagnostic challenges

Myoclonus is a hyperkinetic disorder marked by sudden, brief, involuntary muscle contractions (8, 9). In our case, the patient presented with cervical myelopathy symptoms accompanied by PSM and involuntary vocalizations, which posed a risk of misdiagnosis as epilepsy or psychogenic disorders. The diagnosis of PSM in this case was strictly established based on Brown's clinical diagnostic criteria. Although EEG/EMG data were unavailable due to institutional limitations, the patient's clinical findings perfectly correlated with these established markers: (1) involuntary jerks occurring exclusively in the recumbent position; (2) a flexor-predominant movement pattern; and (3) axial propagation with preserved consciousness. These clinical features, coupled with the immediate postdecompression resolution (<24 h), provide robust diagnostic validation for a spinal origin over cortical or psychogenic etiologies, consistent with established literature (3, 6, 10, 11).

### 3.2 Pathophysiological mechanism

The pathophysiology of PSM in this case is likely centered on the spinal segmental generator (SSG) theory (3–5). Chronic mechanical compression of the cervical cord can impair local inhibitory interneurons, leading to the hyperexcitability of the SSG (6, 10). We hypothesize that the patient's involuntary vocalizations were a specific manifestation of abdominal myoclonus. The rapid recruitment of abdominal and intercostal muscles during myoclonic bursts likely caused sudden, forceful expiration (12). Simultaneously, irritation of the cervical spinal cord at the C3–C5 levels—where the phrenic nerve nuclei reside—could have triggered involuntary diaphragmatic activation. This coordinated but involuntary respiratory muscle contraction, coupled with forced airflow through the vocal cords, resulted in the episodic vocalizations observed (6, 11). This neurophysiological link explains the synchronicity between truncal jerks and sounds, further confirming that the symptoms were purely secondary to structural spinal irritation.

### 3.3 Rationale for endoscopic ACDF

Endoscopic ACDF minimizes tissue trauma, enables precise decompression, and accelerates recovery. In this case, blood loss was only 50 mL, ambulation resumed on postoperative day 1 with brace support, and the drain was removed the same day. Postoperative imaging confirmed stable implants, restored cervical lordosis, and complete decompression (Figure 5). At discharge, tremors and vocalizations had resolved, with marked improvement in lower limb strength, gait stability, mJOA, and Tinetti scores (Table 1) (13).

The immediate resolution of PSM and vocalizations within 24 h postoperatively provides critical diagnostic and pathophysiological insight. This rapid recovery suggests that the symptoms were primarily driven by a “structural compressive etiology,” where the mechanical irritation of the SSG was directly relieved by decompression. In contrast, if long-term compression had induced significant “neuroplastic changes” or central sensitization within the spinal circuits (12), a much more protracted recovery period would typically be expected, as neural pathways require time to remodel and stabilize. Therefore, the near-instantaneous cessation of myoclonic activity in this case serves as robust clinical evidence that the PSM was purely secondary to acute-on-chronic mechanical irritation rather than irreversible maladaptive neuroplasticity.

### 3.4 Comparison with literature

A systematic search (PubMed/Embase, terms: “propriospinal myoclonus” AND “cervical”) identified seven prior surgical PSM cases, all performed using open techniques (Table 2) (6, 10, 11, 13). Although these previous studies reported successful symptom resolution, they lacked granular quantitative metrics regarding perioperative recovery. In contrast, our report provides the first objective evidence of the superior clinical efficiency of endoscopic ACDF, achieving the lowest blood loss (50 mL), fastest ambulation (POD1), and longest recurrence-free follow-up (12 months). In particular, the immediate resolution of symptoms within 24 h provides a direct quantitative contrast to the potentially longer recovery trajectories associated with more invasive open surgeries. While neuroplasticity and nociplastic pain may contribute to chronic sensitization (14), the acute resolution following decompression strongly supports a primary structural compressive mechanism.

**Table 2:** Comparison with previously reported surgical cases of PSM.

Study	Year	Etiology	Level	Surgery
Christodoulides et al. (6)	2018	C5/6 disc herniation	C5/6	Open ACDF
Tapia Perez et al. (10)	2020	Degenerative myelopathy	C4–C6	Laminectomy
Zhang et al. (11)	2025	Cervical spinal cord injury	C4–C5	Conservative
Boudier-Revéret et al. (13)	2020	Postepidural injection	Cervical	Conservative

### 3.5 Limitations and future directions

Limitations of this study include the absence of EEG/EMG and reliance on clinical observation and patient-reported outcomes for PSM characterization. Although the 12-month follow-up showed no recurrence and full functional recovery (resumed manual labor), longer-term studies are needed. Future research should incorporate neurophysiological testing, standardized scales (e.g., Tinetti), and validate endoscopic ACDF in larger PSM cohorts to establish its role in compressive spinal myoclonus.

## 4 Conclusions

Clinically, we emphasize integrating history, examinations, and imaging through multidisciplinary consultations for an accurate diagnosis of complex spinal disorders. This case demonstrated successful endoscopic minimally invasive surgery for multi-segmental SM, with a VAS reduction from 6 to 1, highlighting the importance of recognizing atypical symptoms (e.g., vocalizations). The findings represent the first reported use of endoscopic ACDF for PSM.

## Data Availability

The original contributions presented in the study are included in the article/Supplementary Material, further inquiries can be directed to the corresponding author/s.
